# *Thlaspi arvense* suppresses gut microbiota related TNF inflammatory pathway to alleviates ulcerative colitis

**DOI:** 10.3389/fimmu.2025.1537325

**Published:** 2025-04-22

**Authors:** Wenkai Wang, Yiyang Zhao, Ziwei Wang, Chaowei Wang, Ling Bi, Yan Wang

**Affiliations:** ^1^ Department of Oncology, Shuguang Hospital Affiliated to Shanghai University of Traditional Chinese Medicine, Shanghai, China; ^2^ The Second Clinical Medical College of Guizhou University of Traditional Chinese Medicine, Guizhou, China

**Keywords:** *Thlaspi arvense* (TA), ulcerative colitis (UC), TNF-α, inflammatory pathway, NF-κB, gut microbiota

## Abstract

**Introduction:**

Thlaspi arvense (TA), commonly known as “Ximi” or “Subaijiang,” is a traditional Chinese medicinal herb used to prevent and treat ulcerative colitis (UC). However, the precise mechanisms underlying its therapeutic effects remain unclear, necessitating further investigation to identify potential pharmaceutical applications for UC management. This study aims to elucidate the efficacy and mechanisms of TA and its active constituents in UC treatment.

**Methods:**

This study first evaluated the effects of varying TA doses on 3% dextran sulfate sodium (DSS)-induced UC. Gut microbiota alterations in UC mice were analyzed via 16S rRNA sequencing, with correlation analyses to reveal the relationship between gut microbiota and cytokines. Then, network pharmacology was utilized to identified potential TA targets for UC treatment. Protein-protein interaction (PPI) networks, Gene Ontology (GO), and Kyoto Encyclopedia of Genes and Genomes (KEGG) enrichment analyses were employed to explore TA’s mechanisms. Molecular docking and dynamics simulations validated interactions between TA’s active compounds and UC-related targets. Finally, TNF pathway modulation by TA and its active component, isovitexin, was verified *in vitro* and *in vivo*.

**Results:**

TA alleviated DSS-induced weight loss in a dose-dependent manner, reduced disease activity indices, and preserved intestinal mucosal barrier integrity. Subsequently, fluorescence *in situ* hybridization (FISH) revealed TA suppressed microbial translocation in intestinal tissues. To further characterize inflammatory responses, ELISA demonstrated that TA modulated levels of key cytokines (TNF-α, IL-1β, IL-6, IL-10) and oxidative stress markers (SOD, MDA), indicating systemic anti-inflammatory effects. Building on these findings, 16S rRNA sequencing analyses showed that TA regulated gut microbiota alpha/beta diversity and inhibited infectious disease-related pathways. Notably, correlation heatmaps highlighted a strong association between TNF-α levels and *Escherichia-Shigella* abundance, with high-dose TA significantly reducing this pathogenic bacterial genus. To systematically explore molecular mechanisms, network pharmacology identified 220 potential TA targets for UC treatment. Consistent with experimental data, PPI and KEGG analyses implicated TNF-α, IL-6, and AKT as key targets, primarily through TNF signaling pathway modulation. To validate these predictions, molecular docking confirmed stable interactions between TA compounds and identified targets, while dynamics simulations specifically emphasized isovitexin’s high affinity for TNF-α. Finally, experiments *in vivo* demonstrated TA’s inhibition of TNF-α-mediated NF-κB pathway activation, and *in vitro* studies confirmed that isovitexin directly mitigated TNF-α-induced intestinal epithelial damage. Furthermore, TA demonstrated potent inhibition of TNF-α-mediated NF-κB inflammatory pathway activation in intestinal tissues, while its active constituent isovitexin effectively mitigated TNF-α-induced epithelial cell damage, collectively highlighting their complementary anti-inflammatory mechanisms.

**Discussion:**

Collectively, Thlaspi arvense (TA) ameliorates ulcerative colitis through synergistic mechanisms involving gut microbiota modulation, inflammatory pathway suppression, and intestinal barrier preservation. By remodeling microbial communities to reduce *Escherichia-Shigella* colonization and microbial translocation. TA concurrently inhibits TNF-α/NF-κB-driven inflammation, and oxidative stress regulation. Furthermore, its active constituent isovitexin directly attenuates TNF-α-induced epithelial damage, demonstrating multi-scale therapeutic efficacy. These findings establish TA’s multi-target pharmacology spanning host-microbe interactions and intracellular signaling, while providing a rationale for standardizing TA-based formulations and advancing isovitexin as a precision therapeutic agent for inflammatory bowel diseases.

## Graphical Abstract

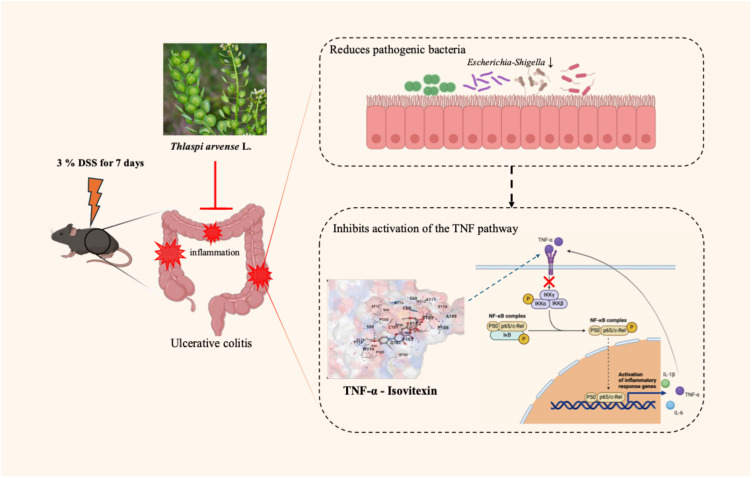

## Introduction

Inflammatory bowel disease (IBD), encompassing Crohn’s disease and ulcerative colitis(UC), significantly compromises patient quality of life and health outcomes. UC manifests as chronic inflammation of the colonic and rectal mucosa/submucosa, with lesions ranging from localized rectal involvement to pancolonic manifestations. Clinically, UC is associated with reduced life expectancy, elevated colectomy risks, and heightened colorectal cancer susceptibility (1, 2). Epidemiologic studies reveal a rapid escalation in UC hospitalization rates across emerging industrialized nations since the early 21st century, particularly in Asian regions. Global UC prevalence is projected to exceed 5 million cases by 2023, with rising incidence rates worldwide imposing growing burdens on healthcare systems (3, 4).

Ulcerative colitis exhibits a biphasic clinical course characterized by acute exacerbations and chronic remission states. During acute flares, patients typically present with hallmark symptoms including profuse diarrhea, abdominal cramping, and hematochezia. Current therapeutic strategies employ agents such as mesalazine(5-aminosalicylic acid, 5-ASA) derivatives and corticosteroids for induction therapy, while maintenance regimens incorporate 5-ASA compounds, thiopurines, biologics (e.g., anti-cytokine/anti-integrin agents), and small-molecule inhibitors targeting Janus kinase or sphingosine-1-phosphate receptors. Nevertheless, 10-20% of refractory UC cases ultimately require proctocolectomy (2). Given the increasing global utilization of complementary medical systems, notably Traditional Chinese Medicine and Ayurveda, there is a pressing need to develop evidence-based complementary therapies for UC prevention and management.

Traditional Chinese Medicine demonstrates distinct therapeutic advantages in managing inflammatory bowel disease and gastrointestinal disorders, particularly through its efficacy and favorable safety profiles (5, 6). *Thlaspi arvense* (TA), commonly termed “Ximi” or “Subaijiang” in Traditional Chinese Medicine, refers to the dried aerial parts of this species native to Eurasian regions, TA serves dual roles as a medicinal resource and a promising oilseed crop with agricultural potential (7) (8). Pharmacological investigations have validated the anti-inflammatory and antioxidant capacities of TA, showing clinical benefits in mitigating chronic pelvic inflammatory disease and hyperuricemia through compound formulations (9, 10). Phytochemical analyses further identify key bioactive constituents, including flavonoids, alkaloids, and polyunsaturated fatty acids, that confer antimicrobial, anti-inflammatory, and redox-modulating properties (11, 12).

Intestinal microbiota dysbiosis constitutes a pivotal etiological factor in ulcerative colitis pathogenesis. This microbial imbalance aligns with the observed therapeutic effects of natural compounds in modulating host-microbe interactions. Compelling evidence demonstrates that UC patients exhibit diminished gut microbial α-diversity compared to healthy controls, with characteristic overgrowth of inflammation-driving pathogens such as *Candida* spp. and *Escherichia coli* that directly contribute to mucosal barrier impairment and inflammatory responses (13–15). The compromised mucosal barrier facilitates pathogen infiltration into submucosal layers, triggering immune cell activation and pro-inflammatory cytokine release. This cascade initiates a self-perpetuating cycle of epithelial damage and disease progression (14). Current therapeutic strategies employ antimicrobial agents like berberine hydrochloride during acute flares (16), while probiotic supplementation has emerged as adjunct therapy for chronic UC management and remission maintenance (17, 18).

Historically, Thlaspi arvense has been employed in polyherbal formulations such as the classical prescription YiYi-FuZi-BaiJiang San, demonstrating therapeutic efficacy against UC and colorectal cancer in both experimental studies and clinical trials (19–21). Notwithstanding these empirical applications, the mechanistic basis of TA’s anti-UC activity remains inadequately characterized. To elucidate the mechanism of *Thlaspi arvense* in ulcerative colitis, we hypothesize that TA ameliorates UC through dual regulatory mechanisms: restoring gut microbial homeostasis to suppress pathogenic bacterial proliferation, and inhibiting TNF-α-dominated inflammatory cascades, thereby preserving intestinal barrier integrity.

To systematically deconvolute the therapeutic mechanisms and bioactive constituents of *Thlaspi arvense* (TA) in ulcerative colitis (UC) management, we employed a tripartite investigative framework. Firstly, network pharmacology analysis identified potential TA-UC interaction targets, complemented by molecular docking simulations to quantify binding affinities between TA phytochemicals and core disease-related proteins. Subsequently, experimental validation of prioritized signaling pathways elucidated TA’s regulatory effects on inflammatory cascades. Parallelly, 16S rRNA sequencing was integrated to delineate TA-induced modulations in gut microbial ecology, thereby bridging host-microbe crosstalk with mucosal immunity. This multimodal approach not only deciphers TA’s multi-target pharmacology but also establishes a translational paradigm for botanical drug development in inflammatory bowel diseases.

## Methods

### Extraction and quality control of TA

The dried aerial parts of TA were purchased from the Chinese pharmacy of Shuguang Hospital, affiliated with Shanghai University of Traditional Chinese Medicine (origin: Anhui, batch number: 20230513, supplied by Shanghai Yanghetang Chinese Herbal Pieces Co., Ltd.). Following the preparation method described by Wang et al. (22), 1 kg of TA was soaked in 8 L of purified water for 1 hour. The mixture was heated to boiling using an electric ceramic kettle and maintained at a boil for 30 minutes. The decoction was filtered through eight layers of gauze to obtain Filtrate A. This extraction process was repeated with 6 L of purified water to obtain Filtrate B. Filtrates A and B were combined and concentrated using rotary evaporation at 60°C. The concentrated solution was lyophilized to produce a freeze-dried powder, yielding 15.34% (w/w). The freeze-dried powder was stored in a cool, dry place, protected from light. For analysis, the freeze-dried powder was reconstituted in 10 mL of 80% methanol and analyzed using UPLC-MS/MS (chromatographic parameters, mass spectrometry conditions, and methodological details are provided in Supplementary Information S1).

### Reagents and chemicals

Dextran sulfate sodium (DSS) with a molecular weight range of 36-50 kDa was supplied by MP Biomedicals Inc. (Solon, Ohio, USA). The positive control drug, sustained-release mesalazine granules (5-aminosalicylic acid), was purchased from Shuguang Hospital affiliated with Shanghai University of Traditional Chinese Medicine (Shanghai Ai De Pharmaceutical Co., Ltd., batch number 231018). ELISA kits for mouse total superoxide dismutase (SOD, JL12237), malondialdehyde (MDA, JL53632), interleukin-1 beta (IL-1β, JL18442), interleukin-6 (IL-6, JL20268), tumor necrosis factor-α (TNF-α, JL10484), and interleukin-10 (IL-10, JL20242) were procured from Shanghai Jianglai Biotechnology Co., Ltd. Sinigrin (RFS-H05311805017), isovitexin (RFS-Y11602204022), orientin (RFS-H04411803007), apigenin (RFS-Q00211801029), and luteolin (RFS-M02501909016) were obtained from Chengdu Refiney Biotechnology Co., Ltd. Antibodies to GAPDH, tubulin, NF-κB, p-NF-κB, IκB, IKKα/β, and p-IKKα/β were provided by Cell Signaling Technology (Massachusetts, USA), while antibodies to caspase-1, ZO-1, claudin-1, and p-IκB were supplied by Proteintech (Wuhan, China).

### Animal studies

Forty 7-week-old C57BL/6J mice, each weighing 20 g, were randomly assigned to five groups (eight mice per group). The study comprised five groups: (1) Control group (Control): Received daily oral gavage of sterile water (vehicle) without DSS induction; (2) DSS model group (Model): Administered 3% DSS water + daily vehicle gavage; (3) Positive control group (5-ASA): 3% DSS + mesalazine (200 mg/kg/day, i.g.); (4) TA low-dose group (TA-L): 3% DSS + TA extract (2.3 g/kg/day, i.g., a clinical equivalent dose); (5) TA high-dose group (TA-H): 3% DSS + TA extract (4.6 g/kg/day, i.g.). After a 3-day pretreatment period with oral gavage, mice received 3% DSS in drinking water, with the DSS solution replaced every 48 hours, for a total DSS exposure duration of 7 days.

### Cell culture

NCM460 cells were maintained in Dulbecco’s Modified Eagle Medium (DMEM, L110KJ, BasalMedia Co., Ltd.) supplemented with 10% fetal bovine serum (FBS, v/v), 100 U/mL penicillin, and 100 μg/mL streptomycin under standard culture conditions (37°C, 5% CO2, humidified atmosphere).

### Cell counting kit-8 assay

The digested cells were resuspended in DMEM and seeded into a 96-well plate at a density of 50,000 cells/mL, with 100 µL per well. The outermost wells were filled with an equal volume of phosphate-buffered saline (PBS) to minimize edge effects. The plate was incubated for 6 hours to allow cell attachment. After incubation, the original medium was removed, and the cells were treated with DMEM containing different concentrations of isovitexin for 24 or 48 hours. Following treatment, the medium was discarded, and 100 µL of DMEM containing 10% (v/v) CCK-8 reagent was added to each well. The plate was incubated in the dark for 30 minutes, and after which the absorbance was measured at 450 nm using a microplate reader. Cell viability was calculated based on the optical density (OD) values, with untreated cells serving as the control.

### *In vitro* models

To evaluate the protective effects of isovitexin on intestinal epithelial cells, NCM460 cells were allocated into different experimental groups (n=3 replicates/group): (1) Control group: cells maintained in standard culture conditions; (2) Model group: cells treated with 10 ng/mL recombinant human TNF-α for 24 hours; (3) Co-treatment with TNF-α (10 ng/mL) and isovitexin at 25 μM, 50 μM, or 100 μM in DMEM. Post-treatment, cells were harvested for downstream analyses including immunofluorescence staining, Western blotting, and quantitative real-time PCR (qPCR; primer sequences provided in **Supplementary Table S1**).

### Disease activity index assessment

Throughout the experiment, the mice were weighed daily at a fixed time, and their fecal characteristics were observed and recorded. Fecal occult blood tests were performed to assess fecal blood content. The Disease Activity Index (DAI) was assessed based on percentage body weight loss, stool consistency, and fecal blood score, with averaged scores determined according to Wang et al. (23). This scoring system provided a preliminary evaluation of model establishment and treatment efficacy.

### Histopathological evaluation

After 14-day of drug administration, mice were euthanized. The distal colon (1 cm proximal to the anus) was excised, rinsed with PBS to remove residual feces, and fixed in 4% paraformaldehyde. Following paraffin embedding, the intestinal tissue was serially sectioned into 4 µm thick slices. Hematoxylin and eosin (H&E) staining was performed to evaluate histopathological changes, with scoring conducted according to Elkholy et al. (24). Alcian blue-periodic acid-Schiff (AB-PAS) staining was utilized to quantify goblet cells, and cell counts were analyzed using ImageJ software.

### Immunofluorescence staining

Paraffin-embedded intestinal tissue sections were deparaffinized with xylene and rehydrated via graded ethanol. Antigen retrieval was performed by microwave heating in citrate buffer (pH 6.0). After permeabilization with 0.25% Triton X-100, sections were blocked with 5% BSA for 1 h at room temperature. Dual labeling was achieved by incubating with ​ZO-1 antibody (Cy3 conjugate, 1:200) and ​Claudin-1 antibody (FITC conjugate, 1:200) ​overnight at 4°C. Post-washing with PBS, nuclei were counterstained with DAPI. Slides were mounted with anti-fade reagent and stored at 4°C in the dark prior to imaging using a laser-scanning confocal microscope.

### Fluorescence *in situ* hybridization

Intestinal tissue sections were deparaffinized with xylene (3 × 15 min) and rehydrated through a graded ethanol series (100%, 95%, and 70%, 5 min each). Antigen retrieval was performed by microwave heating in 1× citrate-based antigen retrieval buffer (pH 6.0) at high power until boiling, followed by low-power maintenance for 15 min to restore epitope accessibility. Sections were then pre-hybridized with probe-free hybridization buffer at 38°C for 1 h in a humidified chamber to block nonspecific binding. The universal bacterial probe EUB338 (5′-GCTGCCTCCCGTAGGAGT-3′) was denatured at 95°C for 5 min, immediately chilled on ice, and hybridized to tissue sections at 38°C for 8 h in the same hybridization buffer. Post-hybridization, sections underwent stringent washes with preheated 2× SSC (46°C, 30 min) and 2× SSC/0.1% NP-40 (46°C, 30 min) to eliminate unbound probes. Nuclei were counterstained with DAPI, and slides were mounted with anti-fade reagent for fluorescence preservation. All specimens were stored at 4°C in the dark prior to imaging using a laser-scanning confocal microscope.

### Identification of bioactive components in TA and UC therapeutic targets

Using the Python 3.5.1 program, chemical components and targets of Thlaspi arvense (TA) were retrieved from SymMap (http://www.symmap.org/) and ETCM (http://www.tcmip.cn/ETCM/index.php/) databases with search terms “Thlaspi arvense,” “Su Baijiang,” or “Ximi”. Additional phytochemical data were extracted from CNKI and PubMed literature. Potential component targets were predicted via SwissTargetPrediction (http://www.Swisstarget-prediction.ch/), TargetNet (http://targetnet.scbdd.com/), and SEA (https://sea.bkslab.org/) using Python’s pandas and Scrapy modules. UC-related targets were acquired from DisGeNet database (https://www.disgenet.org/) and the GeneCards database (https://www.genecards.org/) using “ulcerative colitis” as the query. Duplicate targets were removed, and gene symbol standardization was performed using NCBI Batch Entrez (https://www.ncbi.nlm.nih.gov/sites/batchentrez) platform.

### Identification of core therapeutic targets for TA in UC

To identify key therapeutic targets of TAin UC, potential targets of TA’s bioactive components were intersected with UC-associated targets using the R package VennDiagram. Overlapping targets were identified and visualized through a Venn diagram to delineate shared mechanisms.

### Construction of protein-protein interaction network and core target screening

The key targets for TA against ulcerative colitis were uploaded to the STRING database (v11.5; https://string-db.org), with Homo sapiens as the organism. The full STRING network was generated using a high-confidence interaction score threshold (≥0.700) and medium false discovery rate (FDR <5%). The resultant PPI network was exported in TSV format and analyzed in Cytoscape (v3.10.0), where core targets were identified through degree centrality analysis and visualized. GO and KEGG Enrichment Analyses Gene Ontology (GO) and Kyoto Encyclopedia of Genes and Genomes (KEGG) pathway analyses were performed for TA’s anti-UC targets using the bioinformatics platform (https://www.bioinformatics.com.cn/) (25, 26). Results were visualized through bubble plots created with R’s ggplot2 package (v3.4.4) (27).

### Molecular docking validation

SDF files of TA’s major bioactive components were retrieved from PubChem (https://www.ncbi.nlm.nih.gov/pccompound). Core targets were selected based on PPI network analysis (Section 1.6.4), KEGG pathway enrichment, and literature evidence. High-resolution (≤2.5 Å) protein structures were acquired from PDB (https://www.rcsb.org/) and Uniprot database (http://www.uni-prot.org/). Molecular docking was performed using AutoDock Vina 2.0 (Scripps Research Institute, USA) with ligand SDF files and protein PDB structures.

### Molecular dynamics simulation of TNF-α binding to isovitexin

A 100 ns molecular dynamics (MD) simulation of the TNF-α-isovitexin complex was performed using GROMACS 2022. The CHARMM36 force field were applied to the protein (28), while GAFF2 parameters were used for ligand topology. The complex was solvated in a cubic box with TIP3P water molecules (29) under periodic boundary conditions. Long-range electrostatic interactions were calculated via the particle-mesh Ewald (PME) method, with neighbor searching managed by the Verlet algorithm. System equilibration included 100,000-step NVT and NPT ensembles (0.1 ps coupling time, 100 ps each). Van der Waals and Coulomb interactions used a 1.0 nm cutoff. Production simulations ran for 100 ns at 300 K and 1 bar.

### Detection of intestinal inflammation and oxidative stress markers

Intestinal tissue was minced and homogenized in lysis buffer supplemented with 1% phosphatase/protease inhibitor cocktail for 30 min. The homogenate was centrifuged at 12,000 rpm for 10 min, and the supernatant was collected for protein concentration quantification. Levels of oxidative stress markers (SOD, MDA) and inflammatory cytokines (TNF-α, IL-1β, IL-6, IL-10) were quantified using species-specific ELISA kits per manufacturers’ protocols.

### Western blotting

Intestinal tissue lysates were centrifuged, and protein concentrations were quantified. Samples were mixed with 5× Laemmli buffer, denatured by boiling (95°C, 5 min), and resolved via SDS-PAGE (10% gel). Proteins were electrophoretically transferred onto 0.44 μm PVDF membranes. Membranes were incubated overnight at 4°C with primary antibodies against: p-NF-κB p65 (1:1000), NF-κB p65 (1:1000), p-IKKα/β (1:1000), IKKα/β (1:1000), p-IκBα (1:1000), IκBα (1:1000), GAPDH (1:1000), cleaved caspase-3 (1:1000), and β-tubulin (1:1000) in TBST containing 5% BSA. After TBST washes, membranes were incubated with HRP-conjugated goat anti-rabbit IgG secondary antibody (1:5000) for 1 hr at 25°C. Protein bands were visualized using ECL substrate and quantified by ImageJ software.

### 16S rRNA V3-V4 region microbiome sequencing

Genomic DNA was extracted from fecal samples, and the hypervariableV3-V4 regions of bacterial 16S rRNA gene were amplified using specific primers. Sequencing libraries were prepared by ligating Illumina adapters to purified amplicons, followed by paired-end sequencing on an Illumina MiSeq platform. Sequencing libraries were prepared by ligating Illumina adapters to purified amplicons, followed by paired-end sequencing on an Illumina MiSeq platform. Sequencing libraries were prepared by ligating Illumina adapters to purified amplicons, followed by paired-end sequencing on an Illumina MiSeq platform (http://www.omicsmart.com).

### Data analysis and visualization

Data analysis was conducted using SPSS 26.0 software, and graphs were generated with GraphPad 9.0. Quantitative data are presented as mean ± SD. Student’s *t*-test was used for two-group comparisons, and one-way ANOVA was applied for multi-group analyses. Data violating normality or homogeneity of variance assumptions were analyzed with the Mann-Whitney U test. P < 0.05 was considered statistically significant.

## Results

### Chemical profile of TA

**Figure 1:**
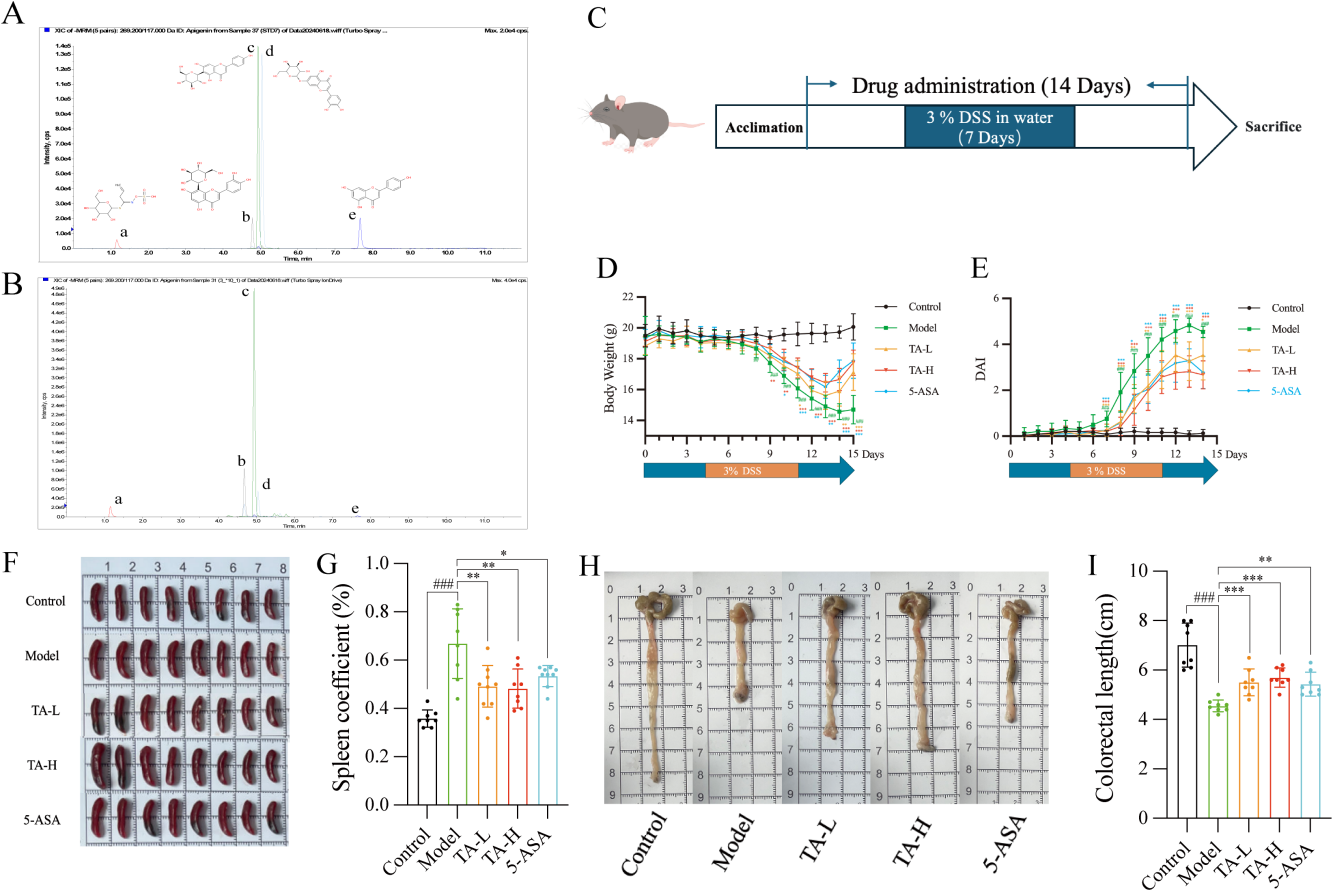
TA alleviates DSS-Induced UC in Mice. **(A)** TIC Chromatograms of 5 representative components standards; **(B)** TIC Chromatograms of 5 representative components in TA (a-Sinigrin, b-Orientin, c-Isovitexin, d-Cynaroside, e-Apigenin); **(C)** Experimental Procedure; **(D)** Changes of body weight in different groups; **(E)** Changes of DAI in different groups; **(F)** Representative spleen images of mice; **(G)** Changes of spleen index in different groups; **(H)** Representative colorectal images of mice; **(I)** Changes of colorectal length in different groups. ^##^ *P* < 0.01, ^###^ *P* < 0.001, compared to Control; *P < 0.05, **P < 0.01, ***P < 0.001, compared to Model.

UPLC-MS/MS quantification of five representative phytochemicals in Thlaspi arvense (TA) revealed distinct compositional patterns (**Figures 1A, B**). Quality-controlled analysis demonstrated the following constituent concentrations per gram of crude TA extract: isovitexin (691.73 μg/g), orientin (636.78 μg/g), sinigrin (439.65 μg/g), cynaroside (47.79 μg/g), and apigenin (12.97 μg/g). These values were calculated based on extraction yield normalization, with isovitexin emerging as the predominant constituent.

### TA ameliorates DSS-induced ulcerative colitis

To investigate the therapeutic potential of TA in DSS-induced ulcerative colitis, with comparative analysis against the standard therapy 5-ASA. 7-day administration of DSS in drinking water was used to induce ulcerative colitis (UC) in mice, with the pharmacological intervention regimen detailed in **Figure 1C**. The results showed that low and high dose TA demonstrated therapeutic effects comparable to 5-ASA. Specifically, TA ameliorated DSS-induced body weight loss from day 9 onward (**Figure 1D**) and reduced disease activity index (DAI) scores (**Figure 1E**). Given that splenomegaly represents a hallmark of systemic inflammation in UC, we quantified the spleen coefficient, calculated as (spleen weight/body weight)×100%, to assess immune status. Following 14 days of treatment, TA significantly attenuated splenomegaly and reduced spleen coefficients (**Figures 1F, G**), paralleling 5-ASA’s efficacy. Furthermore, TA mitigated UC-associated colonic shortening (**Figures 1H, I**), confirming its anti-inflammatory activity comparable to standard therapy.

### TA preserves intestinal barrier integrity in UC

**Figure 2:**
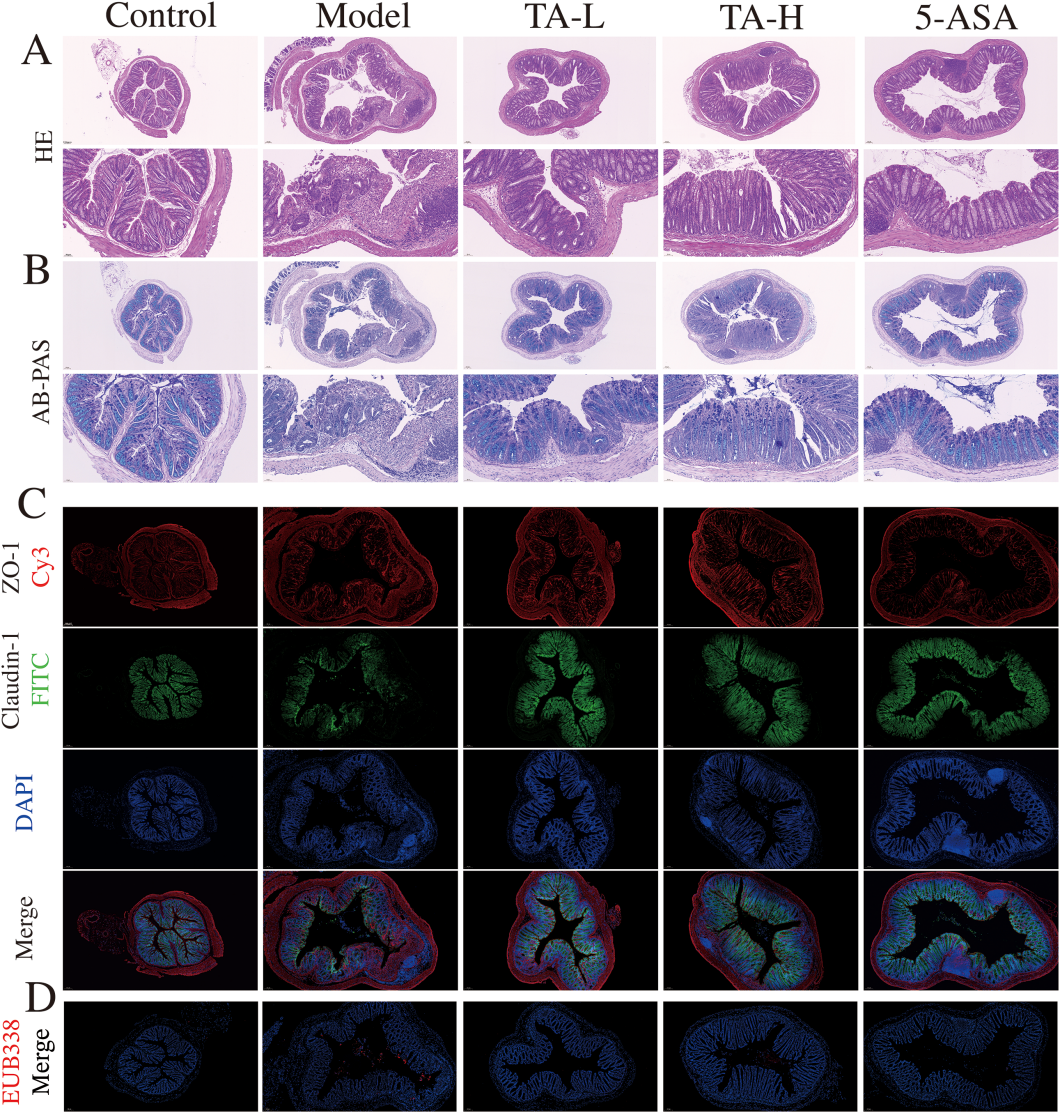
TA alleviates intestinal mucosal injury. **(A)** Histopathological section micrograph (H&E staining, 100× and 200×); **(B)** The content of colon mucus (AB-PAS staining, 100× and 200×); **(C)** ZO-1 and claudin-1 protein expression in the colon (ZO-1- Cy3, claudin-1-FITC, 100×); **(D)** Microbes in intestinal tissue(EUB338-Cy3).

Building on the observed anti-inflammatory effects, we further investigated TA’s capacity to preserve intestinal barrier integrity in DSS-induced colitis. The intestinal barrier, a critical defense mechanism against bacterial translocation and systemic inflammation, demonstrated severe compromise in DSS-treated mice. After 7 days of 3% DSS intervention, mice in the model group exhibited severe intestinal epithelium destruction, crypt architecture loss, and submucosal inflammatory cell infiltration (**Figure 2A**). TA administration significantly attenuated these pathological alterations, with reduced epithelial damage and lower pathological index scores (**Supplementary Material Figure A**). Furthermore, TA treatment markedly increased intestinal goblet cell numbers compared to the model group (**Figure 2B**; **Supplementary Material Figure B**). To evaluate barrier structural integrity, we assessed colonic tight junction proteins, key components of epithelial paracellular sealing. Compared to the control group, DSS-induced colitis significantly reduced ZO-1 and claudin-1 expression. And both TA and 5-ASA treatments effectively restored these critical barrier components to near-normal levels (**Figure 2C**). In recent years, the bacterial translocation due to intestinal mucosal damage has garnered increasing attention. To investigate bacterial translocation, a functional consequence of barrier compromise, we performed fluorescence *in situ* hybridization using the universal 16S rRNA-targeted EUB338 probe (30, 31). The results showed that TA treatment substantially reduced bacterial infiltration into intestinal tissues (**Figure 2D**), confirming its mucosal protective efficacy.

### TA modulates inflammatory and oxidative stress in UC

**Figure 3:**
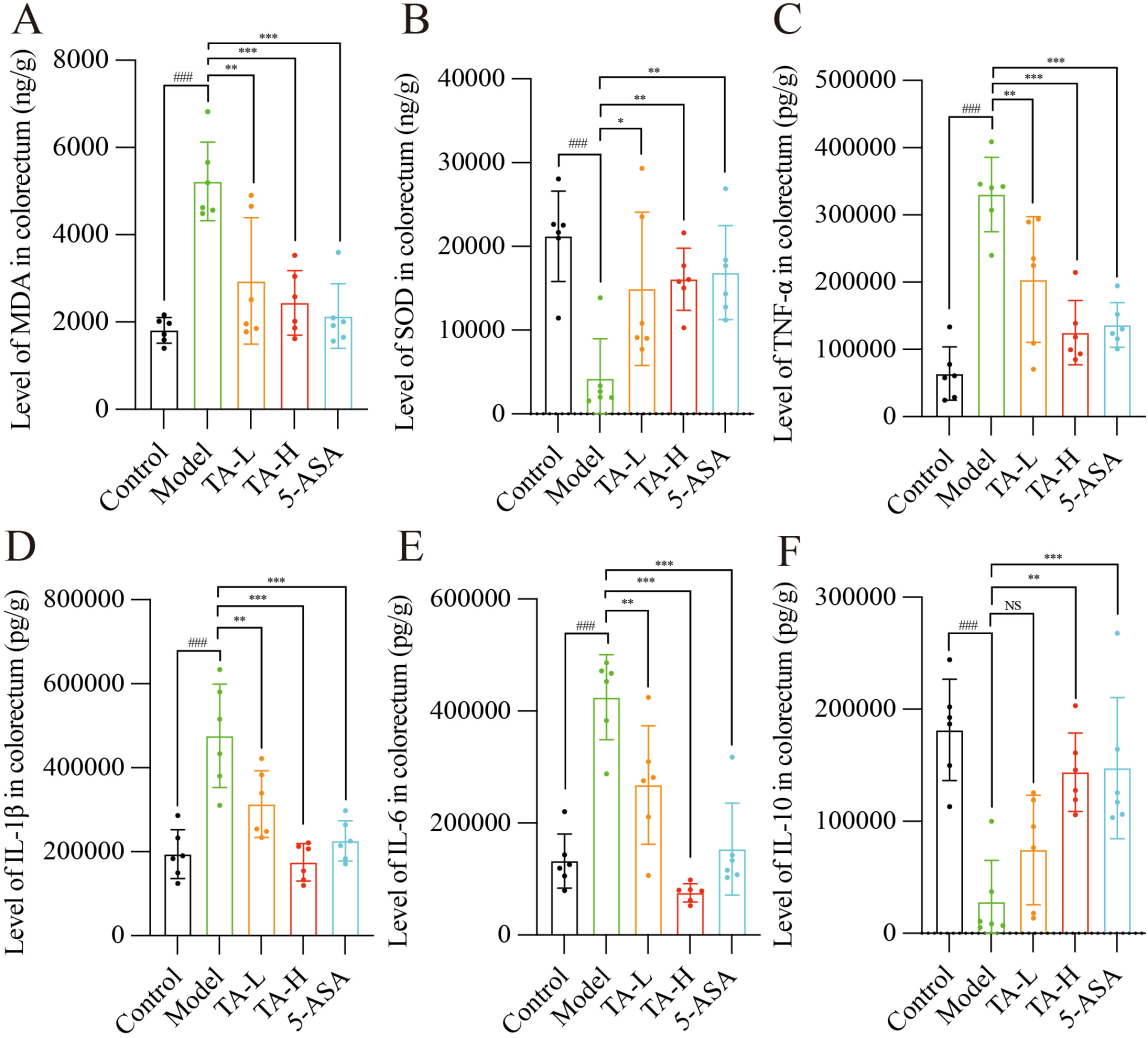
TA reduces the levels of oxidative stress and inflammation in the gut of UC mice. **(A)** The levels of SOD in the intestinal tissues of each group; **(B)** The levels of MDA in the intestinal tissues of each group; **(C)** The levels of TNF-α in the intestinal tissues of each group; **(D)** The levels of IL-1β in the intestinal tissues of each group; **(E)** The levels of IL-6 in the intestinal tissues of each group; **(F)** The levels of IL-10 in the intestinal tissues of each group. ^###^ *P* < 0.001, compared to Control; *P < 0.05, **P < 0.01, ***P < 0.001, NS, P>0.05, compared to Model.

To delineate the anti-inflammatory potential of TA beyond structural barrier repair, we quantified oxidative stress and cytokine profiles. As illustrated in **Figures 3A, B**, DSS exposure induced significant oxidative imbalance, with MDA levels significantly elevated and SOD activity markedly reduced (P < 0.005) compared to controls. Notably, TA administration dose-dependently ameliorated these alterations. Concurrently, the inflammatory cascade manifested through upregulated pro-inflammatory mediators, including tumor necrosis TNF-α, IL-6, and IL-1β (P < 0.005). TA treatment significantly attenuated these cytokine elevations. (**Figures 3C–E**). Furthermore, the anti-inflammatory cytokine IL-10, which was significantly decreased in the model group (P < 0.005), showed restoration following TA intervention (**Figure 3F**).

### TA modulates gut microbiota composition in colitis

**Figure 4:**
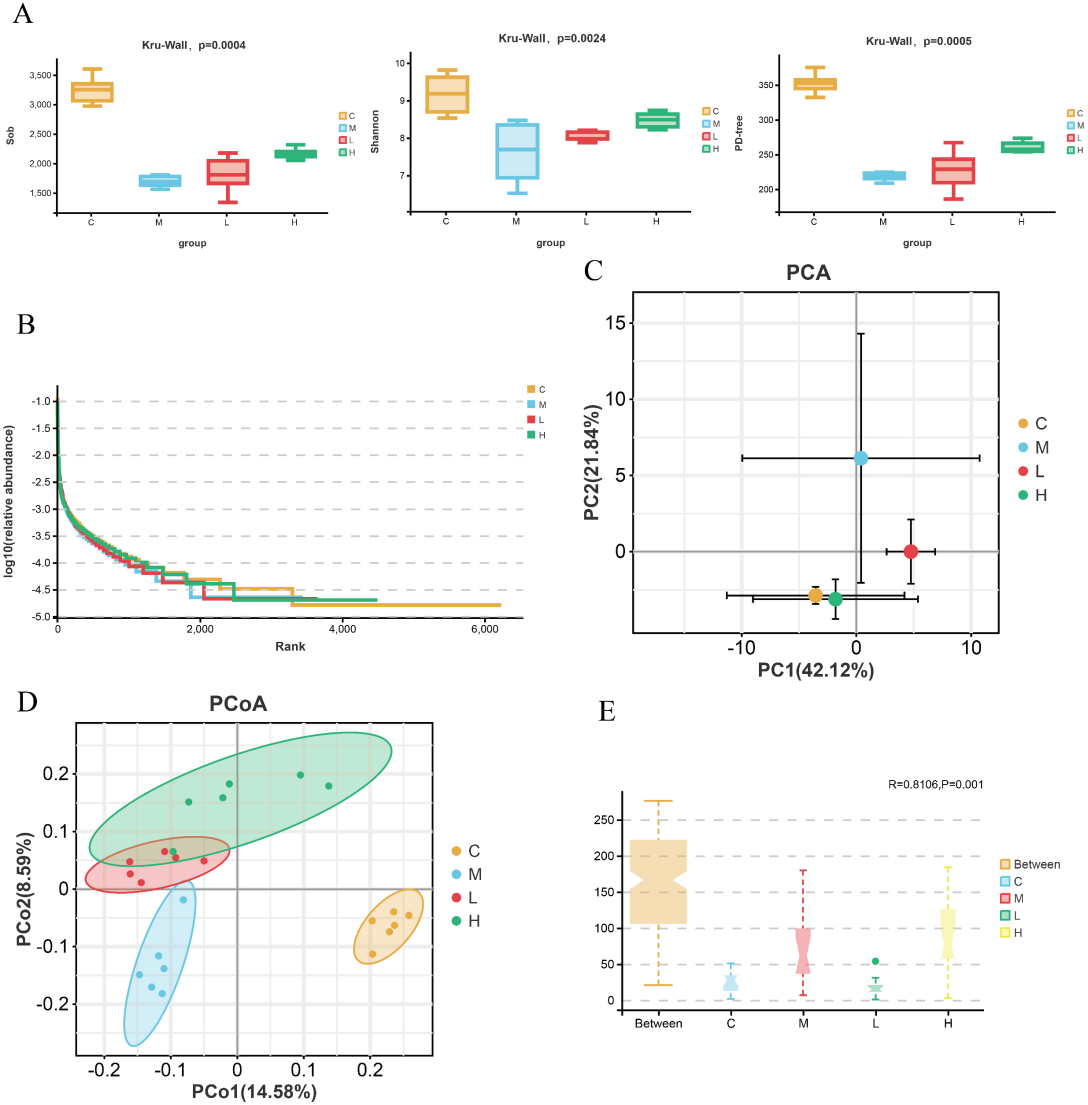
TA regulates the α and β diversity of the mouse gut microbiota. **(A)** Alpha diversity index of gut microbiota in each group(sob, Shannon, PD-tree); **(B)** Rank-abundance curve of the intestinal microbiota of mice; **(C, D)** PCA and PCoA of intestinal microbiota of mice; **(E)** Analysis of similarities (ANOSIM) of intestinal microbiota of mice(distance between samples: unweighted_unifrac; C-Control, M-Model, L-TA-L, H-TA-H).

Given the established role of gut microbiota in intestinal homeostasis, we investigated whether TA-mediated improvements correlate with microbial modulation. As demonstrated in **Figure 4A**, DSS-induced colitis significantly reduced microbial richness (Sob index), whereas TA treatment effectively restored this parameter. Furthermore, TA administration enhanced microbial evenness (Shannon index) and species diversity compared to the model group, with taxonomic distribution shifts visualized in **Figure 4B**. To assess community structural changes, multivariate analyses (PCA/PCoA) revealed distinct clustering patterns: the model group microbiota deviated markedly from controls, while TA intervention partially normalized this dysbiosis (**Figures 4C, D**). Importantly, ANOSIM validation confirmed intergroup dissimilarity (R = 0.816, P = 0.001; **Figure 4E**).

**Figure 5:**
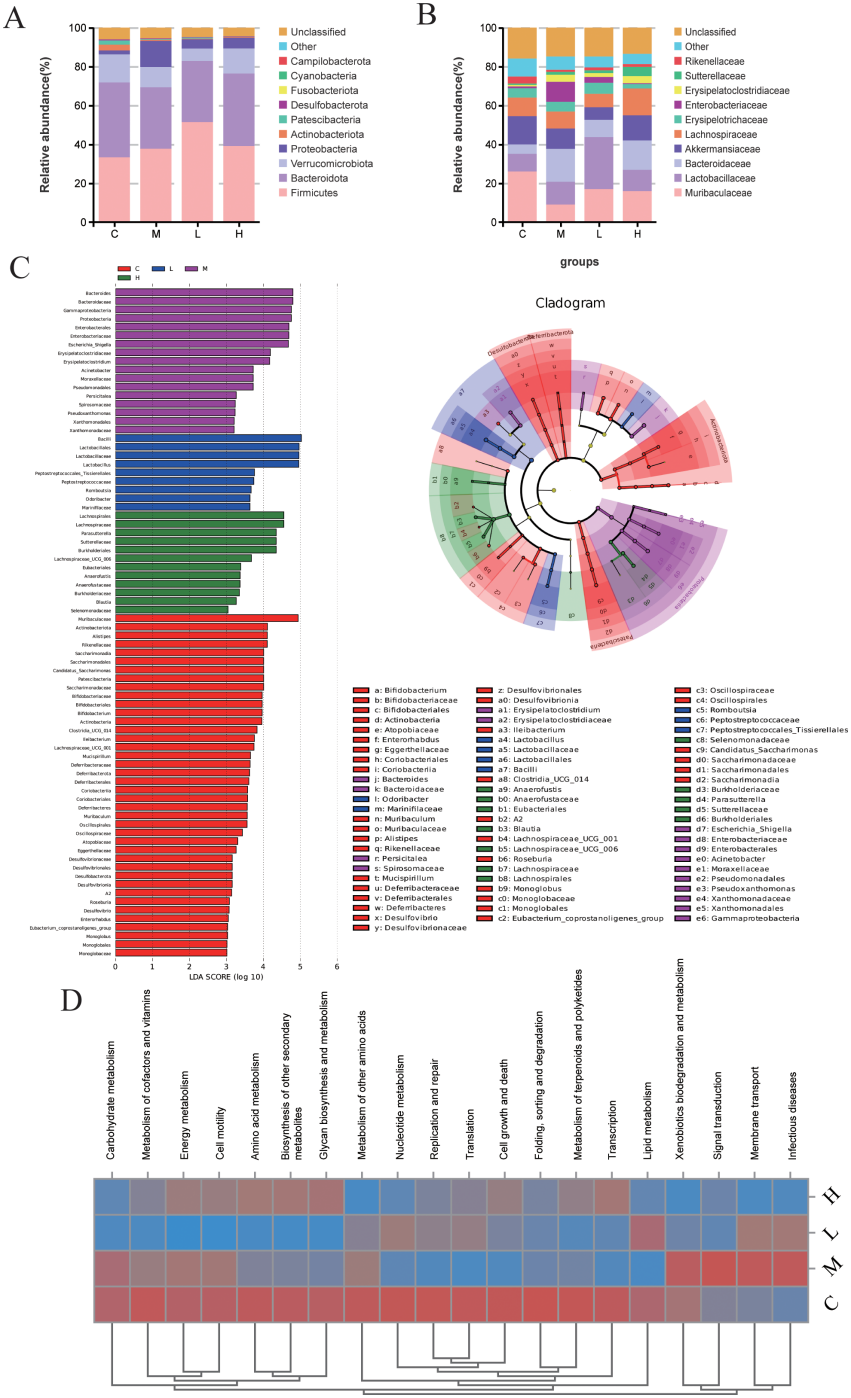
TA regulates the main composition and function of intestinal bacteria in UC mice. **(A)** Phylum-level microbiota in each group; **(B)** Genus-level microbiota in each group **(C)** LEfSe analysis of intestinal microbiota in each group (LDA >3, *P* < 0.05); **(D)** The KEGG heat map of microbiota based PICRUSt2. (C-Control, M-Model, L-TA-L, H-TA-H).

At the taxonomic level, TA suppressed pathobiont expansion, specifically reducing Proteobacteria phylum and Enterobacteriaceae genus abundances (**Figures 5A, B**). Through LEfSe analysis (LDA >3, P <0.05), Muribaculaceae and *Alistipes* were identified as control-associated symbionts, in contrast to model-enriched pathogens (*Escherichia-Shigella*, *Acinetobacter*). Notably, TA dosage influenced commensal selection: *Lactobacillus* dominated low-dose regimens, whereas Lachnospiraceae typified high-dose treatment (**Figure 5C**). Complementing taxonomic alterations, PICRUSt2 prediction associated TA with downregulated pathogenic pathways, particularly infectious disease and transmembrane signaling mechanisms (**Figure 5D**).

**Figure 6:**
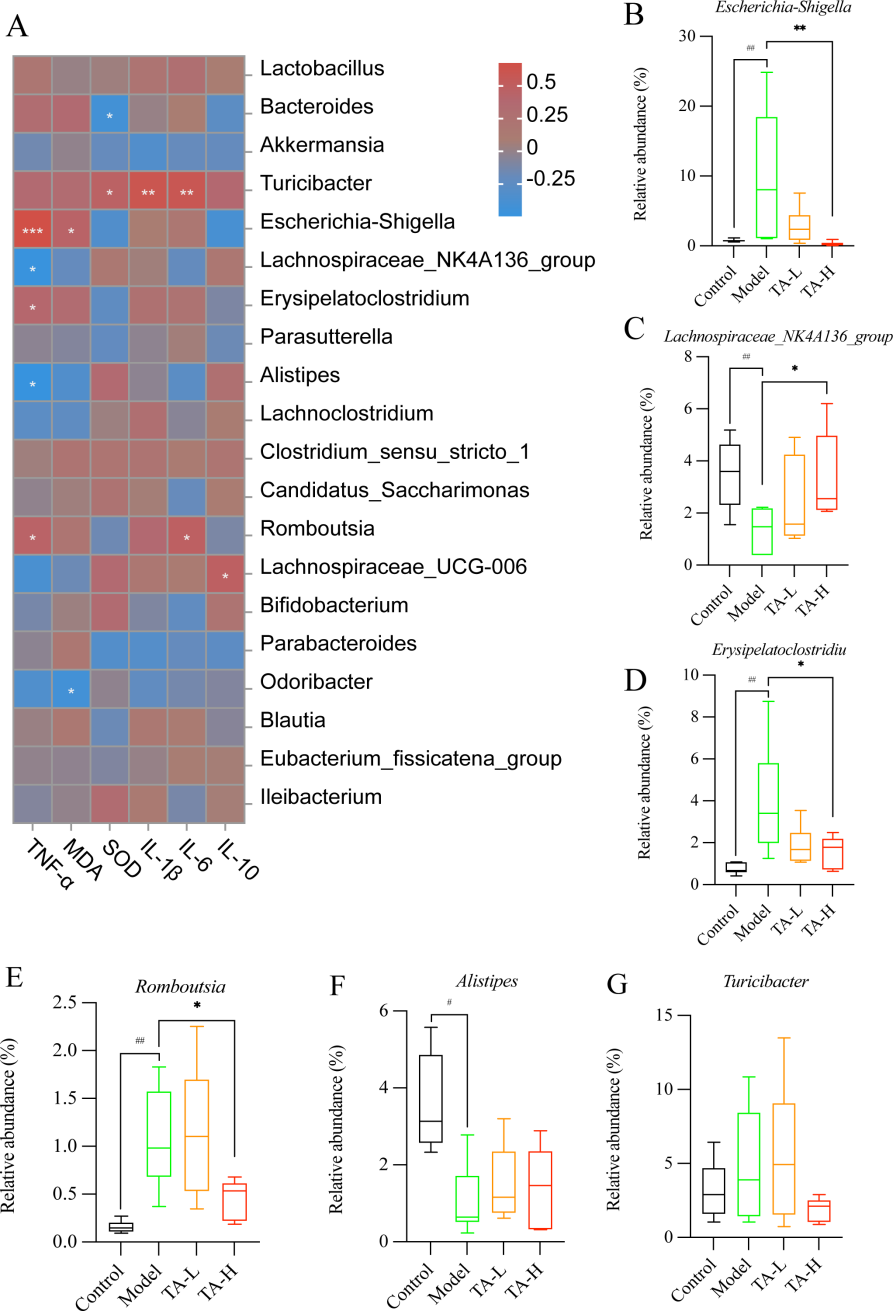
Relationship between gut microbiota and cytokines. **(A)** Heat map of the abundance of gut microbiota in relation to cytokines; **(B–G)**. Genus-level representative microbiota in each group (B-G, Rank sum test, ^#^ *P* > 0.05, ^##^ *P* < 0.01, compared to Control; *P < 0.05, **P < 0.01, ***P < 0.001, compared to Model; C-Control, M-Model, L-TA-L, H-TA-H).

To elucidate host-microbe interactions, Spearman correlation mapped significant bacterium-cytokine relationships (**Figure 6A**). Consistent with pro-inflammatory effects, TA dose-dependently diminished *Escherichia-Shigella* (TNF-α-correlated, P <0.001), *Erysipelatoclostridium*, and *Romboutsia* loads (**Figure 6B, D, E**). Conversely, anti-inflammatory taxa (*Lachnospiraceae_NK4A136_group*, *Alistipes*) exhibited inverse TNF-α associations alongside model group depletion (**Figures 6C, F**). Despite maintaining stable abundance across groups, *Turicibacter* showed correlations with IL-1β, IL-6, and SOD (**Figure 6G**).

### Network pharmacology reveals multitarget mechanisms underlying TA efficacy in UC

**Figure 7:**
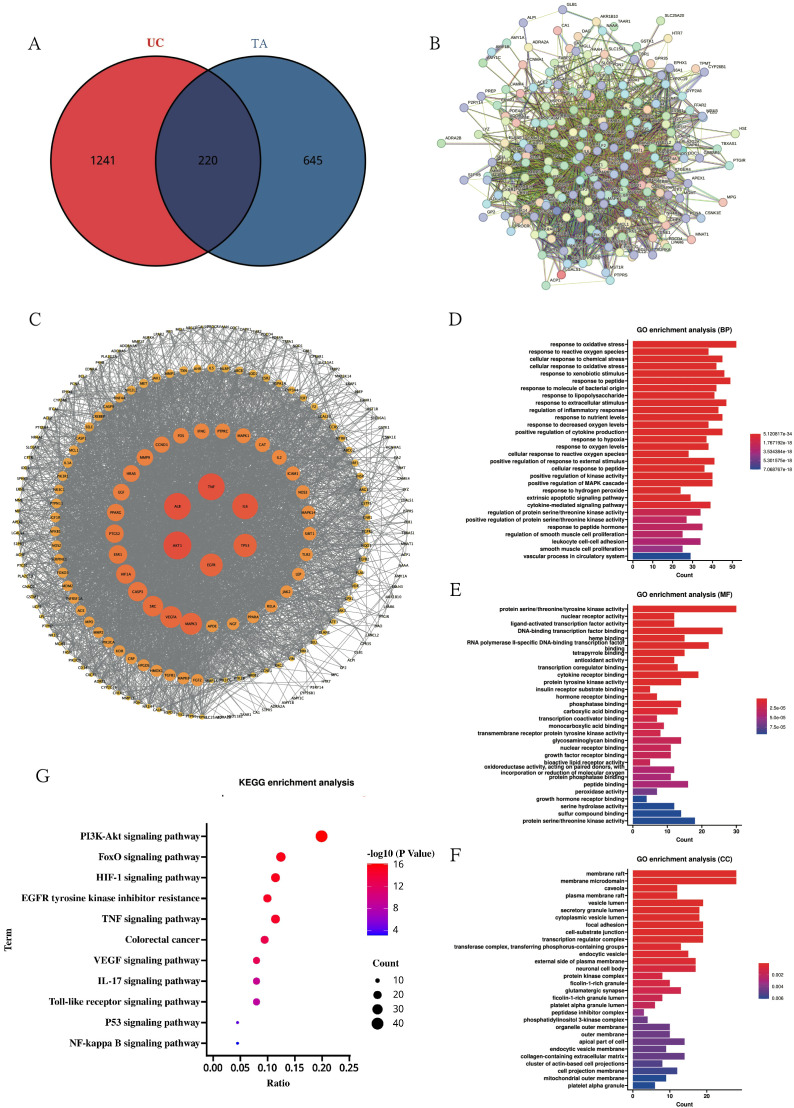
Network pharmacology elucidates the mechanism of TA against UC. **(A)** Venn diagram of UC disease targets and TA active ingredient targets; **(B)** PPI protein interactions network diagram; **(C)** MCODE analysis of key targets; **(D–F)**. GO enrichment analysis of TA in UC(BP diagram; MF diagram; CC diagram); **(G)** KEGG pathway enrichment of *TA* in UC.

With the aim of expounding the molecular basis of the therapeutic effects observed in colitis models, a network pharmacology approach was systematically applied. Target profiling identified 865 bioactive targets associated with TA and 1,461 disease targets related to UC, with 220 overlapping candidates constituting potential therapeutic nodes (**Figure 7A**; **Supplementary Material Tables S2**-**3**). Subsequent PPI network analysis through Cytoscape topology optimization identified TNF, ALB, AKT1, IL-6, TP53, and EGFR as hub targets mediating the anti-colitis effects of TA (**Figures 7B, C**). Functional enrichment analysis revealed three mechanistic dimensions: biological processes involving oxidative stress response and redox homeostasis (**Figure 7D**), molecular functions encompassing kinase regulation and nuclear receptor activation (**Figure 7E**), and cellular localization to membrane microdomains including caveolae (**Figure 7F**). Finally, KEGG pathway mapping further confirmed TNF signaling pathway modulation as the principal mechanism (**Figure 7G**; **Supplementary Material Figure C**), consistent with the cytokine regulatory capacity of TA demonstrated experimentally.

### Molecular docking and dynamics simulations validate TA-target interactions

**Figure 8:**
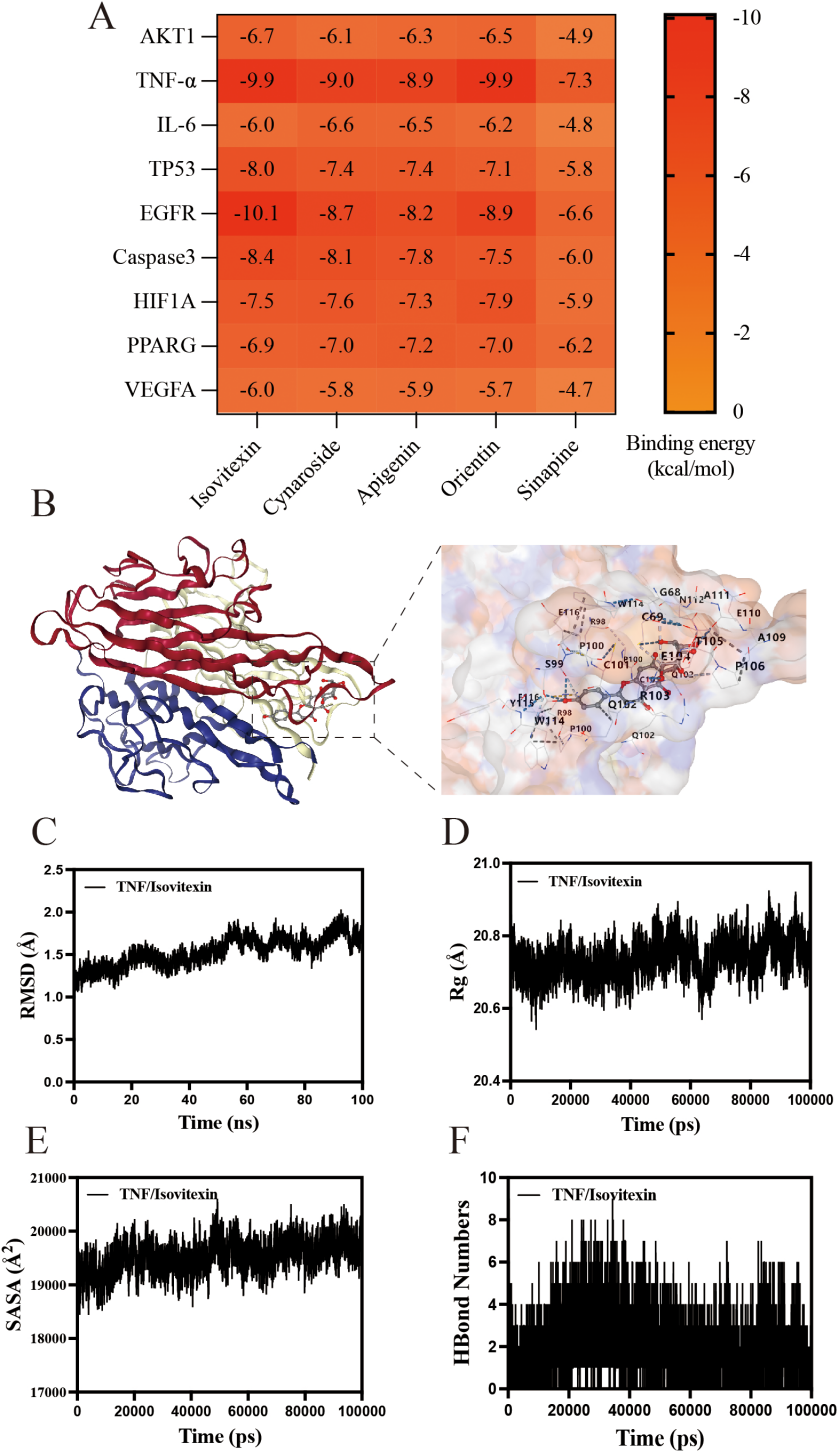
Molecular docking and Molecular Dynamics of components in TA. **(A)** Heatmap of binding energy between TA chemistry and UC target; **(B)** molecular docking of isovitexin to TNF-α; **(C)** The RMSD of isovitexin to TNF-α; **(D)** The Rg of isovitexin to TNF-α; **(E)** The SASA of isovitexin to TNF-α; **(F)** The HBound numbers of isovitexin to TNF-α.

Complementing the network pharmacology findings, molecular docking and dynamics simulations were employed to characterize interactions between TA components and therapeutic targets. Based on PPI and KEGG analyses identifying AKT1 and TNF-α as key mediators, five phytochemicals enriched in TA were computationally docked with nine candidate targets. As shown in **Figure 8A**, the compounds exhibited strong binding affinities to TNF-α, with binding energies below -7.0 kcal/mol. Isovitexin, the predominant constituent identified through quantitative chemical analysis, demonstrated optimal binding geometry with TNF-α (**Figure 8B**). Molecular dynamics simulations further validated complex stability through conformational equilibrium attainment in the isovitexin-TNF-α system, evidenced by root mean square deviation (RMSD) values stabilizing below 2 Å (**Figure 8C**). Radius of gyration (Rg) analysis revealed minimal structural fluctuations (20.462 Å - 20.925 Å), confirming maintained protein compactness (**Figure 8D**). Solvent-accessible surface area (SASA) variations indicated microenvironmental adaptations during ligand binding (**Figure 8E**). Crucially, dynamic hydrogen bond formation (0–9 bonds) between isovitexin and TNF-α (**Figure 8F**) substantiated robust molecular interactions underlying the therapeutic efficacy of TA.

### Therapeutic suppression of inflammatory signaling by TA and isovitexin

**Figure 9:**
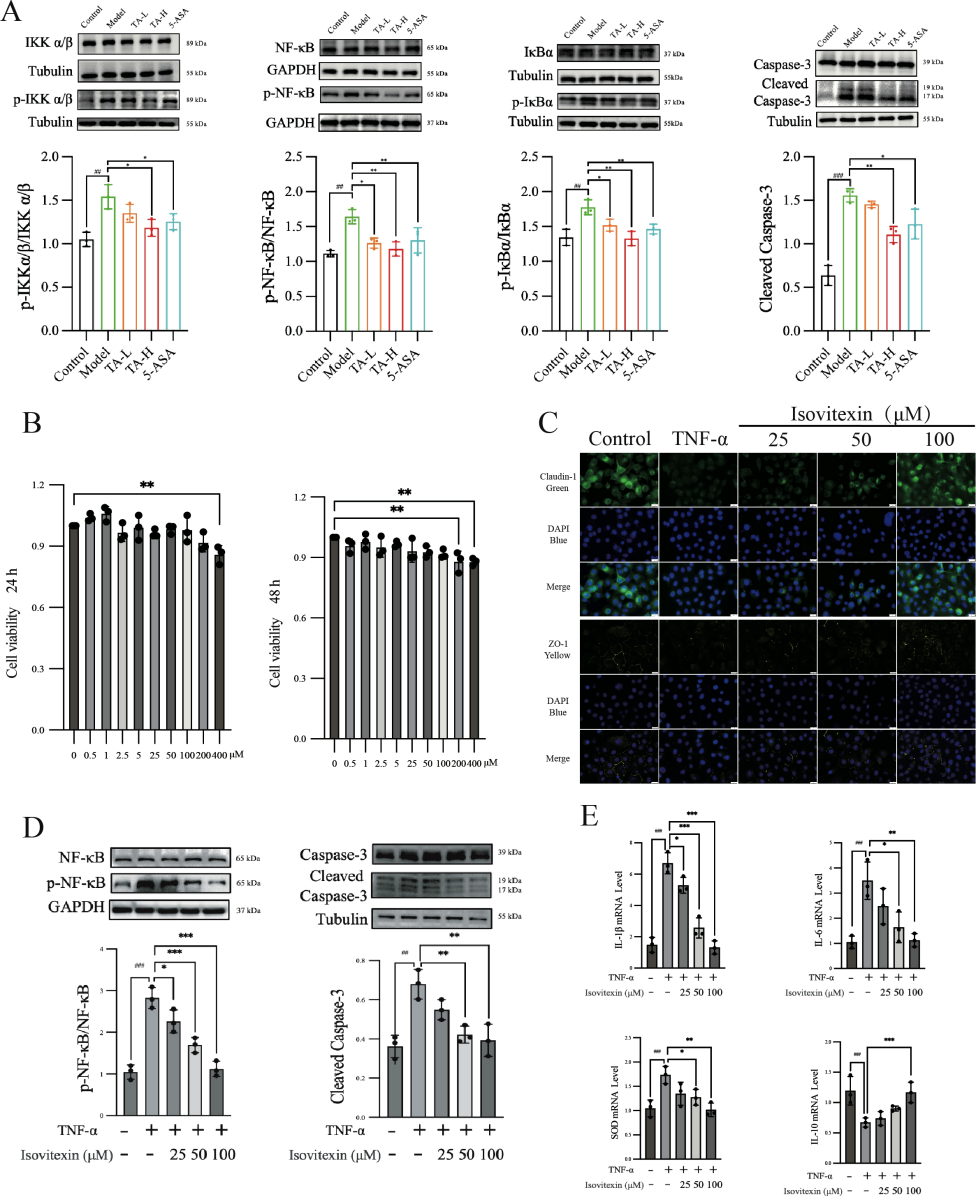
The effects of TA and isovitexin on TNF-mediated UC *in vitro* and *in vivo.* **(A)** WB analysis of TNF-related inflammatory and apoptotic pathway proteins in mouse intestinal tissues; **(B)** CCK-8 assay showing the effect of isovitexin on the viability of NCM460 cells; **(C)** Immunofluorescence analysis the effect of isovitexin on the expression of tight junction proteins; **(D)** Effect of isovitexin on the inflammatory and apoptotic pathway proteins; **(E)** Effect of isovitexin on the mRNA levels of inflammatory cytokines. ^##^ *P* < 0.01, ^###^ *P* < 0.001, compared to Control; *P < 0.05, **P < 0.01, ***P < 0.001, compared to Model(TNF-α).

To clarify the molecular interaction evidence, the anti-inflammatory mechanisms of TA were further investigated through TNF-α signaling pathway analysis in colonic tissues. The results showed that high-dose TA administration significantly suppressed phosphorylation ratios of IKKα/β (p-IKKα/β/IKKα/β), IκB (p-IκB/IκB), and NF-κB (p-NF-κB/NF-κB) compared to model controls (P < 0.01; **Figure 9A**). Concomitant with NF-κB signaling inhibition, both TA and 5-ASA significantly reduced cleaved-Caspase-3 expression in colonic tissues compared to the model group (**Figure 9A**). This suppression of caspase-dependent apoptosis paralleled the anti-inflammatory efficacy observed with clinical standard therapy, confirming the functional linkage between NF-κB pathway inhibition and apoptosis regulation in colitis management. Complementing these *in vivo* observations, the principal bioactive constituent isovitexin identified in TA was systematically evaluated for therapeutic efficacy *in vitro*.

Initial cytotoxicity assessment using CCK-8 assay revealed that 400 μM isovitexin reduced NCM460 cell viability within 24 hours, with 200 μM exhibiting cytotoxic effects after 48-hour exposure (**Figure 9B**). In parallel, immunofluorescence imaging demonstrated that TNF-α treatment (24 hours) significantly diminished Claudin-1 and ZO-1 expression in intestinal epithelial cells. Notably, isovitexin co-treatment concentration-dependently restored tight junction protein localization (**Figure 9C**). Mechanistic interrogation further showed that 100 μM isovitexin effectively suppressed TNF-α-induced phosphorylation of NF-κB (p-NF-κB/NF-κB ratio) and caspase-3 activation (cleaved-Caspase-3), with statistical significance (P < 0.01; **Figure 9D**). Transcriptional profiling via qPCR confirmed dose-responsive regulation of TNF-α-dysregulated genes, including pro-inflammatory mediators (IL-1β, IL-6), anti-inflammatory cytokine IL-10, and antioxidant enzyme SOD (**Figure 9E**).

## Discussion

The pathogenesis of ulcerative colitis (UC), characterized by chronic intestinal inflammation with clinical manifestations including diarrhea, hematochezia, and weight loss, was effectively recapitulated in DSS-induced murine model. As a sulfated polysaccharide derivative, DSS disrupts epithelial integrity and induces microbiota dysbiosis, mirroring human UC pathophysiology. Consistent with established protocols (32), 3% DSS administration induced elevated disease activity index (DAI), splenomegaly, and colorectal shortening, aligning with established UC biomarkers (33–35). These pathological features collectively confirm the validity of the DSS-induced UC model for therapeutic evaluation.

The therapeutic efficacy of TA extends beyond symptom relief to fundamental barrier restoration, as demonstrated by its administration via oral gavage markedly alleviating UC manifestations including diarrhea resolution, reduced hematochezia, and attenuated weight loss, accompanied by significant DAI reduction. As a classical Chinese medicinal herb first documented in the Shennong Materia Medica as “Su Baijiang” (classified as a superior-grade herb), TA has been traditionally used in Jiangsu, Zhejiang, and Hubei provinces for managing gastrointestinal disorders such as dysentery and enteritis (36). The intestinal mucosal barrier, comprising four interdependent components (biological, chemical, mechanical, and immune barriers), showed DSS-induced disruption of its chemical barrier through significant reductions in goblet cells and mucin secretion. Both TA and 5-aminosalicylic acid (5-ASA) effectively reversed these pathological changes (37, 38). Mucin secreted by goblet cells serve dual functions in microbiota regulation and pathogen exclusion while maintaining intestinal sterility (39–41). The mechanical barrier, formed by epithelial tight junctions and bacterial membranes, prevents microbial translocation into systemic circulation (42). DSS administration significantly decreased expression of tight junction proteins ZO-1 and Claudin-1, which are crucial for maintaining paracellular transport regulation and epithelial integrity (43, 44). TA treatment restored these protein levels comparably to 5-ASA. These findings collectively demonstrate TA’s dual therapeutic action through symptom alleviation and mucosal barrier repair.

The gut microbiota maintains homeostasis through stable composition and spatial organization, forming an interdependent micro-ecosystem that constitutes the intestinal biological barrier (45, 46). Our experimental data revealed decreased α-diversity in the DSS-induced model group compared to controls, indicating reduced microbial richness, abundance, and ecological balance. This finding aligns with clinical observations of dysbiosis patterns in ulcerative colitis patients (47). These observations collectively demonstrate successful replication of UC-associated microbial imbalance in our experimental model.

In addition, our analysis revealed significant enrichment of *Escherichia-Shigella* in UC group mice, reaching 10-20% abundance compared to 1% in healthy controls. This pathogenic expansion mirrors clinical reports showing elevated *Escherichia-Shigella* levels (10-20%) during active UC compared to normal human levels (1%) (37), with established mechanistic links to tight junction disruption in UC patients (48). Further supporting these observations, LEfSe analysis identified *Acinetobacter* as a prominent UC-associated taxon, consistent with findings of bloodstream *Acinetobacter* detection in UC patients (49). Complementing these microbial findings, the therapeutic potential of TA manifested through significant suppression of *Escherichia-Shigella* proliferation, corroborated by PICRUSt2 functional prediction analysis suggesting antimicrobial effects through infectious disease-related pathways. And quantitative chemical analysis revealed high sinigrin content in TA (439.65 μg/g), a compound recognized for antimicrobial precursor potential (50), with its metabolite allyl isothiocyanate demonstrating specific antibacterial activity against *Escherichia coli* (51). This antimicrobial mechanism may involve microbial glycoside hydrolase-mediated metabolism of glucosinolates enhancing production of isothiocyanate, thiocyanate, and nitrile compounds, thereby inhibiting pathogenic growth *(* 52 *)*.

For antimicrobial mechanisms, network pharmacological analysis provided critical molecular insights. As an emerging approach for investigating complex herbal systems, network pharmacology has demonstrated particular utility in traditional Chinese medicine research (53, 54). Our study identified TNF-α as the principal therapeutic target of TA through systematic screening, with KEGG pathway analysis confirming significant enrichment in TNF-α signaling, a pathway of established clinical relevance given elevated TNF-α levels in UC patients and ongoing development of TNF-α inhibitors like adalimumab (55). Mechanistic investigation revealed that TNF-α activates downstream inflammatory pathways including NF-κB and NLRP3, ultimately promoting apoptosis through coordinated signaling. The canonical NF-κB pathway operates via sequential activation: TNF-α stimulation triggers IKK complex activation, which phosphorylates IκB proteins. This phosphorylation liberates the NF-κB/Rel complex, enabling its nuclear translocation where it independently or cooperatively with transcription factors drives expression of pro-inflammatory cytokines such as IL-1β and IL-6 (56). Having systematically identified TNF-α as the principal therapeutic target of TA, molecular docking demonstrated strong spontaneous binding (ΔG = -9.9 kcal/mol) between TNF-α and the two predominant constituents in TA - isovitexin and orientin. This high-affinity interaction mechanistically explains the observed inhibition of downstream NF-κB signaling, particularly the suppression of IL-1β-mediated caspase activation. Specifically, activation of the NF-κB pathway elevates IL-1β levels, which facilitate cleavage of pro-caspase-3 into apoptosis-executing Cleaved Caspase-3 (57). Treatment with TA effectively blocked this proteolytic cascade by attenuating NF-κB activation mediated through TNF-α.

Notwithstanding the mechanistic insights gained, this study presents limitations necessitating consideration. First, although the antimicrobial effects of TA against pathogenic bacteria including *Escherichia-Shigella* were conclusively demonstrated, the specific bioactive components responsible for this inhibition remain unidentified. While the whole-herb preparation of TA shows significant microbiota-modulating capacity, precise identification of the active antimicrobial constituents requires future isolation and characterization through component-specific assays such as agar diffusion testing with inhibition zone quantification. Second, the observed correlation between microbiota modulation and inflammation alleviation requires causal validation to distinguish direct microbial regulation from secondary metabolite-mediated effects. Planned investigations employing fecal microbiota transplantation combined with metabolomic profiling will elucidate whether the therapeutic benefits derive from specific microbial community changes or their metabolic byproducts. Collectively, these methodological innovations establish a systems pharmacology framework that synergizes phytochemical analysis with microbial dynamics, creating an evidence-based paradigm to decode the therapeutic mechanisms of bioactive constituents in traditional Chinese medicine while elevating global scientific recognition of herbal pharmacopeia.

## Conclusion

In this study, we employed network pharmacology, molecular docking, and 16S rRNA sequencing, among other methodologies, to comprehensively elucidate the therapeutic effects of *Thlaspi arvense*, a traditional Chinese herbal medicine, on DSS-induced UC. Our findings suggest that the protective mechanisms of TA involve the inhibition of the TNF-α-mediated NF-κB inflammatory pathway, suppression of pathogenic intestinal microbiota proliferation, and maintenance of intestinal mucosal barrier integrity. These results establish a foundational framework for developing TA as a therapeutic agent while highlighting its potential in UC prevention and management.

## Data Availability

The datasets presented in this study are deposited in the NCBI (https://www.ncbi.nlm.nih.gov/) repository, accession number: PRJNA1243376.
