# Supplementary material for: Thlaspi arvense suppresses gut microbiota related TNF inflammatory pathway to alleviates ulcerative colitis
Source: Front Immunol. 2025 Apr 22;16:1537325. doi: 10.3389/fimmu.2025.1537325 (PMC12053237; doi:10.3389/fimmu.2025.1537325)
Supplement: Supplementary file 1 [file DataSheet1.zip › Supplementary Information S1 & Figures.DOCX]

**Sample Processing and Instrument information for Quantitative Analysis:** Add each sample to 80% methanol and bring the volume up to 10 mL. Perform ultrasonic extraction for 40 minutes, allow the sample to cool to room temperature, and then replenish the weight with 80% methanol solution. Centrifuge at 3000 r/min for 5 minutes, filter the supernatant solution through a 0.22 μm membrane, and collect approximately 1 mL of the filtrate. Dilute the filtrate 10 times with 80% methanol. Prepare two parallel samples, each with a 2 μL injection.

Liquid Chromatography: WATERS Ultra Performance Liquid Chromatography (UPLC)

Mass Spectrometry: AB Sciex 6500 Series Mass Spectrometer

Mobile Phase: Acetonitrile with 0.1% Formic Acid, containing 4 mM Ammonium Formate

MRM ACQUISITION SETTINGS OF TARGET COMPOUNDS

| Q1 Mass (Da) | Q3 Mass (Da) | Param |  | ID |
| --- | --- | --- | --- | --- |
| 269.2 | 117 | DP | -200 | Apigenin |
|  |  | CE | -39 |  |
|  |  | CXP | -13 |  |
|  |  |  |  |  |
|  |  |  |  |  |
|  |  |  |  |  |
| Q1 Mass (Da) | Q3 Mass (Da) | Param | Start | ID |
| 358.2 | 97.1 | DP | -45 | Sinigrin |
|  |  | CE | -23.5 |  |
|  |  | CXP | -11 |  |
|  |  |  |  |  |
|  |  |  |  |  |
|  |  |  |  |  |
| Q1 Mass (Da) | Q3 Mass (Da) | Param | Start | ID |
| 431.2 | 311.2 | DP | -160 | Isovitexin |
|  |  | CE | -29 |  |
|  |  | CXP | -11 |  |
|  |  |  |  |  |
|  |  |  |  |  |
|  |  |  |  |  |
| Q1 Mass (Da) | Q3 Mass (Da) | Param | Start | ID |
| 447.2 | 327.1 | DP | -160 | Orientin |
|  |  | CE | -31 |  |
|  |  | CXP | -18 |  |
|  |  |  |  |  |
|  |  |  |  |  |
|  |  |  |  |  |
| Q1 Mass (Da) | Q3 Mass (Da) | Param | Start | ID |
| 447.1 | 285 | DP | -100 | Luteolin |
|  |  | CE | -35 |  |
|  |  | CXP | -15 |  |

The detection settings were as follows: Ion spray voltage, 4500 V; entrance potential, 10 V(-); curtain gas, 241.3 kPa; CAD, 48.3 kPa; source temperature, 500°C; nebulizer gas (gas 1), 413.7 kPa; heater gas (gas 2), 413.7 kPa.

| Gradient Table | |  |  |
| --- | --- | --- | --- |
| Time (min) | Flow Rate（mL/min） | Aqueous phase (%) | Organic phase (%) |
| Initial | 0.3 | 95 | 5 |
| 1.5 | 0.3 | 95 | 5 |
| 3 | 0.3 | 75 | 25 |
| 6 | 0.3 | 65 | 35 |
| 8 | 0.3 | 30 | 70 |
| 9 | 0.3 | 10 | 90 |
| 9.5 | 0.3 | 5 | 95 |
| 12 | 0.3 | 95 | 5 |


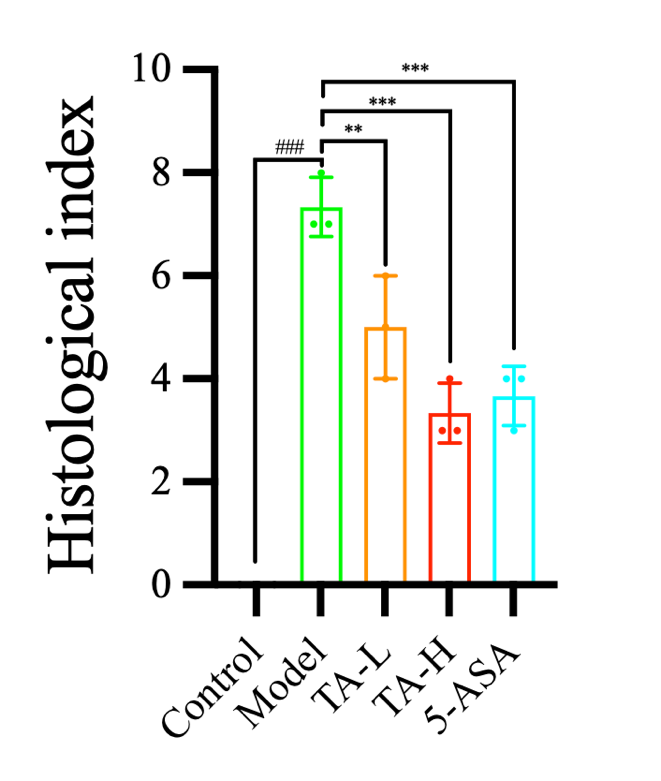


Fig. A Intestinal histopathological score in each group


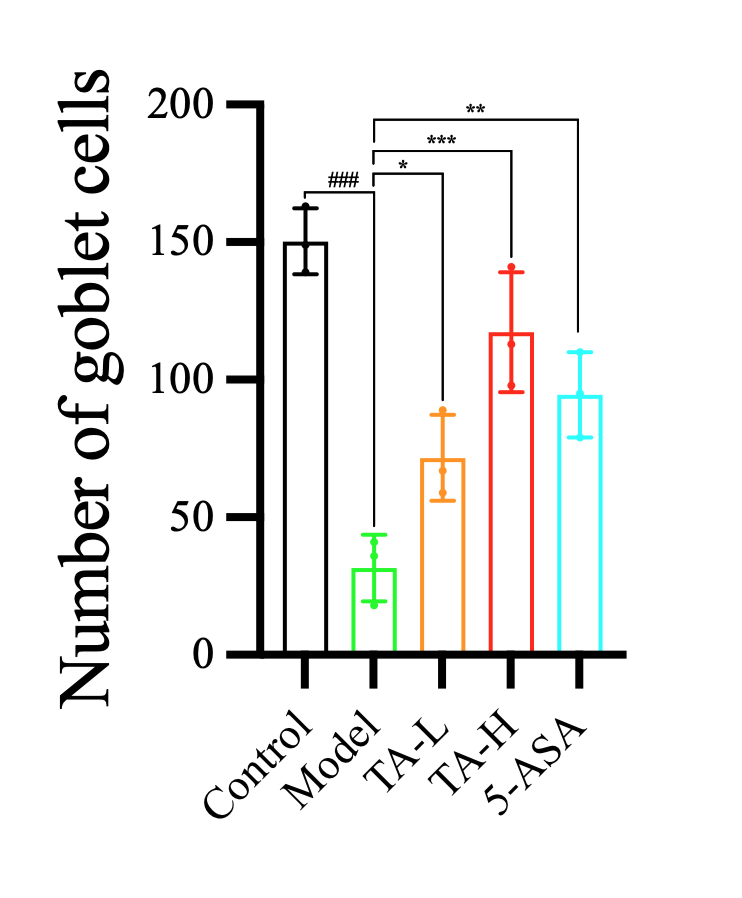


Fig. B The number of intestinal goblet cells in each group


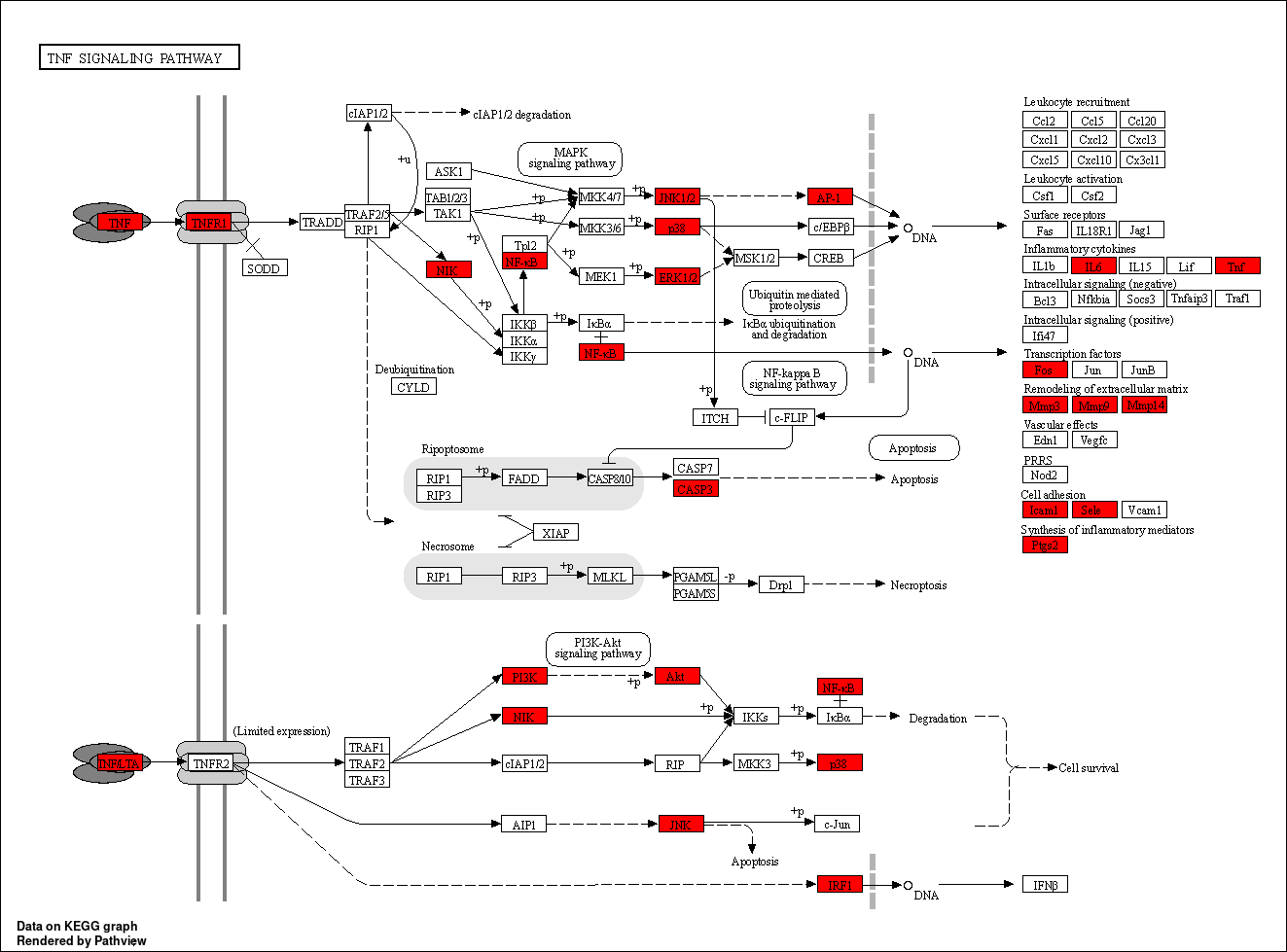


Fig. C The KEGG pathway of TNF
